# Adsorption Performance of Magnetic Covalent Organic Framework Composites for Bisphenol A and Ibuprofen

**DOI:** 10.3390/molecules28135214

**Published:** 2023-07-05

**Authors:** Beibei Zhang, Ye Tian, Xuezhen Gao, Hui Zheng, Yuzhong Niu, Junshen Liu

**Affiliations:** Institute of Environmental Science, School of Chemistry and Materials Science, Ludong University, Yantai 264025, China; tiany1609@163.com (Y.T.); gaoxuez@163.com (X.G.); zhenghui@ldu.edu.cn (H.Z.); niuyuzhong@ldu.edu.cn (Y.N.)

**Keywords:** covalent organic frameworks, magnetic, adsorption, bisphenol A, ibuprofen

## Abstract

As typical environmental endocrine disruptors and nonsteroidal anti-inflammatory drugs, bisphenol A and ibuprofen in water supplies can cause great harm to the ecological environment and human health. In this study, magnetic covalent organic framework composites Fe_3_O_4_@COF-300 were synthesized by the hydrothermal method and used to remove bisphenol A and ibuprofen from water. Fe_3_O_4_@COF-300 could be rapidly separated from the matrix by external magnetic fields, and could selectively adsorb bisphenol A and ibuprofen in the presence of coexisting compounds such as phenol, Congo red, and amino black 10B. The removal efficiency of ibuprofen was 96.12–98.52% at pH in the range of 2–4 and that of bisphenol A was 92.18–95.62% at pH in the range of 2–10. The adsorption of bisphenol A and ibuprofen followed a pseudo-second-order kinetic and Langmuir model, and was a spontaneous endothermic process with the maximum adsorption amounts of 173.31 and 303.03 mg∙g^−1^, respectively. The material presented favorable stability and reusability, and the removal efficiency of bisphenol A and ibuprofen after 5 cycles was still over 92.15% and 89.29%, respectively. Therefore, the prepared composite Fe_3_O_4_@COF-300 exhibited good performance in the adsorption of bisphenol A and ibuprofen in water.

## 1. Introduction

With the rapid development of economies, the content of organic pollutants in the water environment is constantly increasing and gradually becoming an important global problem that threatens the ecological environment and human health. Among these, synthetic organic pollutants are the main source of organic pollutants. These compounds are of an anthropogenic origin from agriculture and industry and can be divided into several main categories according to their origin: pharmaceuticals and personal care products, steroid hormones, surfactants, and industrial chemicals and pesticides [1]. The synthetic organic pollutants generally undergo decomposition at relatively slow rates, and their removal efficiency using traditional sewage treatment methods is low. Thus, organic contaminants entering water bodies pose a serious threat to plant and animal health through bioconcentration [2].

Environmental endocrine disruptors and nonsteroidal anti-inflammatory drugs are two important synthetic organic contaminants derived primarily from industrial chemicals and pharmaceuticals. Environmental endocrine disruptors are substances that can interfere with the endocrine system of humans and other animals and increase the risk of endocrine disorders, leading to reproductive disorders, metabolic diseases, and various cancers. Bisphenol A is a typical environmental endocrine disruptor commonly used in the production of polycarbonate plastics, epoxy linings for canned food and beverage containers, dental sealants, and thermal paper [3]. At present, sewage treatment plants can only remove bisphenol A to a limited extent, so this compound is frequently detected in many water environments. Nonsteroidal anti-inflammatory drugs are widely used, and ibuprofen is one of the most prescribed nonsteroidal anti-inflammatory drugs in the contemporary era. Owing to its extensive global demand, its production reaches more than 30,000 tons per year [4]. The primary source of ibuprofen contamination in the environment is the excretion of nonmetabolic and metabolic drugs in the urine of humans and animals after medical treatment, followed by drug use and disposal in the aquaculture and pharmaceutical industries [5]. Upon reaching our bodies through contaminated water bodies, ibuprofen can induce serious side effects, such as vomiting, gastric ulcers, bleeding, indigestion, intestinal inflammation, mucosal damage, kidney problems, cardiovascular risks, and central nervous system problems [6].

Certain measures must be taken to effectively remove bisphenol A and ibuprofen from natural water bodies and wastewater to avoid their harmful effects on biological health. At present, the main removal methods are adsorption, photocatalysis, advanced oxidation, and biological techniques [7]. Among them, adsorption is widely adopted for the removal of bisphenol A and ibuprofen due to its simplicity and efficiency, as well as repeatability [8]. The performance of an adsorbent medium has an important effect on the economic feasibility of adsorption. Zeolite, activated carbon, silicate, metal–organic frameworks, metal–organic nanotube materials, covalent organic frameworks (COFs), and some other materials are widely used as adsorbents for contaminants owing to their special structure and physicochemical properties [9].

Among these adsorption materials, COFs possess the advantages of adjustable pore size, large specific surface area, diverse synthesis methods, and easy functional modification, and, thus, they are now increasingly used in the field of adsorption. COFs composed of layered stacking of two-dimensional polymers have a large number of ordered pore columns. These pore columns are ideal channels for ion migration and diffusion, which can greatly promote the adsorption of pollutants [10]. The COFs with imine bonds have excellent stability in water bodies, can be used for acid and alkali wastewater treatment, and have the characteristic of preventing secondary pollution [11]. Compared with the simple ones, COFs composited with other materials demonstrate additional physical and chemical properties and are thus extremely popular. For example, a new-generation COF material named SNW–1 was composited with montmorillonite for the removal of anionic and cationic dyes from aqueous solutions [12]. TpBD was composited with graphene oxide for the extraction of bisphenol A [13]. TpPa–1 composited with bimetallic oxide MnFe_2_O_4_ was employed to remove UO_2_^2+^ from aqueous solutions [14]. Especially, Fe_3_O_4_@COF material has many advantages, such as excellent adsorption capacity, significant superparamagnetism, stability, and biocompatibility. These characteristics of Fe_3_O_4_@COF enable rapid separation of materials and matrix in the presence of an external magnetic field, thereby solving the problems of other non-magnetic COFs/composite materials that are difficult to separate from water and difficult to reuse.

In this work, COF-300 was used as substrate due to its mild synthesis conditions, short time consumption, and strong adsorption capacity. The new Fe_3_O_4_@COF-300 composite was prepared and applied for the adsorption of bisphenol A and ibuprofen in water. This study aimed: (1) To prepare and characterize the Fe_3_O_4_@COFs composites; (2) To analyze the effects of solution pH and ionic strength on the adsorption performance of Fe_3_O_4_@COF-300; (3) To explore the adsorption isotherms, adsorption kinetics, thermodynamics, and adsorption mechanism of bisphenol A and ibuprofen by Fe_3_O_4_@COFs.

## 2. Results and Discussion

### 2.1. Characterization of Fe_3_O_4_@COF-300

**Figure 1:**
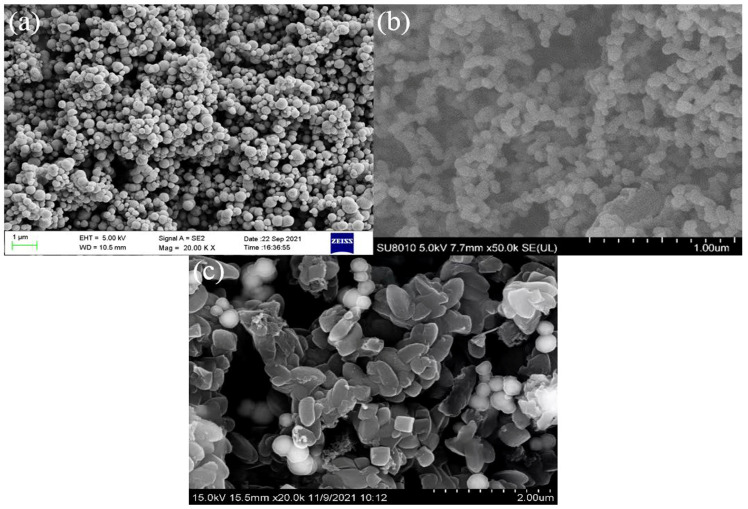
SEM images of Fe_3_O_4_ (**a**), COF-300 (**b**), and Fe_3_O_4_@COF-300 (**c**).

The surface morphology of Fe_3_O_4_, COF-300, and Fe_3_O_4_@COF-300 materials was investigated using scanning electron microscopy (SEM). As shown in Figure 1a,b, Fe_3_O_4_ is a spherical particle with a diameter of approximately 300 nm, and COF-300 is an oval particle, which is consistent with previous literature [15]. Figure 1c demonstrates that many white spherical bright spots are loaded on the surface of COF-300, indicating that Fe_3_O_4_ and COF-300 are successfully compounded.

**Figure 2:**
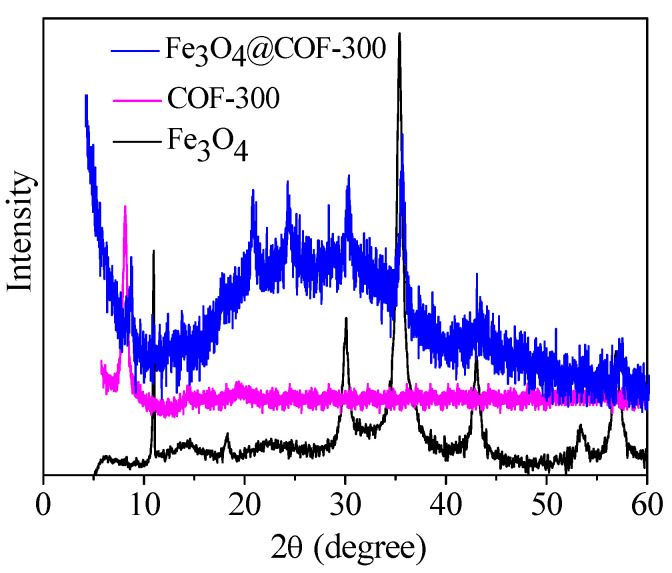
XRD patterns of Fe_3_O_4_, COF-300, and Fe_3_O_4_@COF-300.

Figure 2 shows the X-ray diffraction (XRD) patterns of the Fe_3_O_4_, COF-300, and Fe_3_O_4_@COF-300 samples. A single strong diffraction peak at around 2*θ* = 8.2° was observed for COF-300, indicating that the as-prepared COF-300 possessed high crystallinity. This result was in accordance with that previously reported [16]. Compared with the XRD pattern of COF-300, the main diffraction peaks of Fe_3_O_4_@COF-300 appeared in addition to 2*θ* = 8.6°, another 3 new diffraction peaks located at 2*θ* = 30.3°, 35.3°, and 42.9° corresponding to the (220), (311), and (400) planes of Fe_3_O_4_, respectively [17]. This result indicates the successful preparation of the composites.

**Figure 3:**
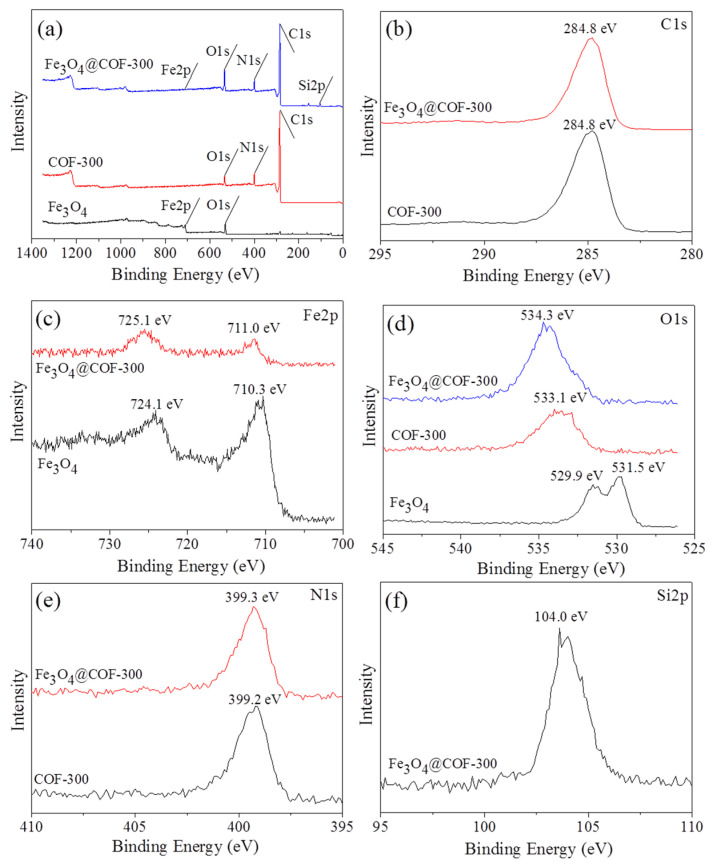
XPS spectra of survey scan of Fe_3_O_4_@COF-300, COF-300, and Fe_3_O_4_ (**a**), C1s (**b**), Fe2p (**c**), O1s (**d**), N1s (**e**), and Si2p (**f**).

The chemical composition and elemental bonding structure of Fe_3_O_4_@COF-300 were explored via X-ray photoelectron spectra (XPS). Figure 3a presents the full-scan XPS spectrograms of Fe_3_O_4_@COF-300, COF-300, and Fe_3_O_4_. The main constituent elements of Fe_3_O_4_@COF-300 include carbon, iron, oxygen, nitrogen, and silicon; those of COF-300 include carbon, oxygen, and nitrogen; those of Fe_3_O_4_ include iron and oxygen. Figure 3b–f illustrate the high-resolution XPS spectrograms of C1s, Fe2p, O1s, N1s, and Si2p. Fe_3_O_4_@COF-300 has a C1s photoelectron peak at 284.8 eV, corresponding to the C=C bond [18]. The high-resolution Fe2p spectrogram of Fe_3_O_4_@COF-300 shows 2 peaks with binding energies of 711.0 and 725.1 eV attributed to the 2p3/2 and 2p1/2 peaks of Fe (III), respectively [19]. An energy of 399.3 eV of Fe_3_O_4_@COF-300 can be assigned to C=N–C bonds, while an energy of 104.0 eV can be attributed to Si–O bonds [20,21]. Fe_3_O_4_ exhibits 2 peaks at 529.9 and 531.5 eV in O1s, attributed to the Fe–O group in Fe_3_O_4_ and OH in water, respectively. However, the peak in Fe_3_O_4_@COF-300 moved to a higher banding energy of 534.3 eV. The reason may be that the existence of the C=O bond in COF-300 could shift the OH in water to the direction of high binding energy [22]. Comparison of high-resolution XPS spectrograms showed that the binding energies of N1s (399.3 eV), O1s (534.3 eV), and Fe2p (711.0 and 725.1 eV) of Fe_3_O_4_@COF-300 are slightly higher than those of COF-300 (N1s: 399.2, O1s: 531.1 eV) and Fe_3_O_4_ (O1s: 529.9 and 531.5 eV, Fe2p: 710.3 and 724.1 eV). These results can be attributed to the interaction of Fe_3_O_4_ with COF-300 that resulted in the inner shift of N1s, O1s, and Fe2p orbits. XPS findings showed that Fe_3_O_4_ and COF-300 are combined by chemical bonds in the prepared composites rather than being simply physically mixed.

**Figure 4:**
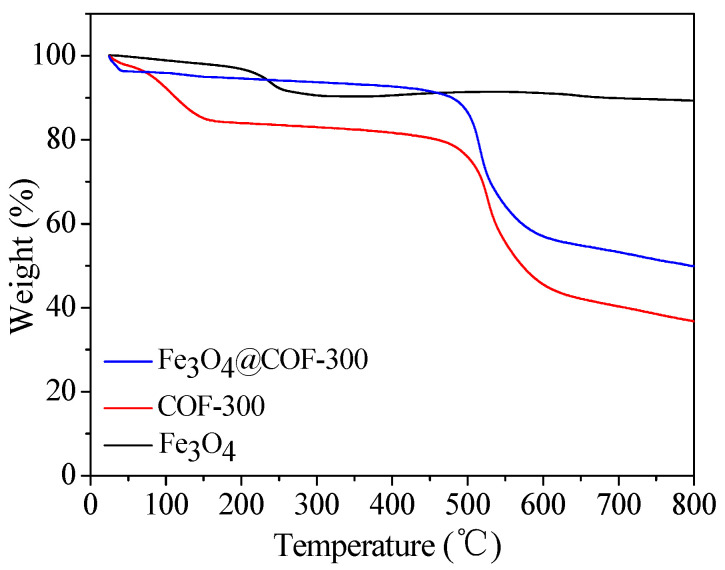
TGA curves of Fe_3_O_4_, COF-300, and Fe_3_O_4_@COF-300.

Thermogravimetric analysis (TGA) of Fe_3_O_4_, COF-300, and Fe_3_O_4_@COF-300 was carried out under nitrogen atmosphere with heating from 25 °C to 800 °C at a heating rate of 10 °C/min. As shown in Figure 4, the initial decrease in Fe_3_O_4_ was mainly due to the volatilization of water on its surface. Meanwhile, the mass loss of COF-300 was 10.0% as the temperature rose to 450 °C. As the temperature continued to rise to 600 °C, the mass loss of COF-300 reached 47.2%. This is mainly due to the fracture of the imine bond and the disintegration of the diamond network structure in the COF-300 material. When the temperature is higher than 600 °C, the mass loss of COF-300 tends to be stable, indicating that the material has been completely decomposed. The TGA curve of Fe_3_O_4_@COF-300 showed a consistent trend with that of COF-300 and lost less than 10% of its weight at 480 °C, indicating that Fe_3_O_4_@COF-300 has good stability.

**Figure 5:**
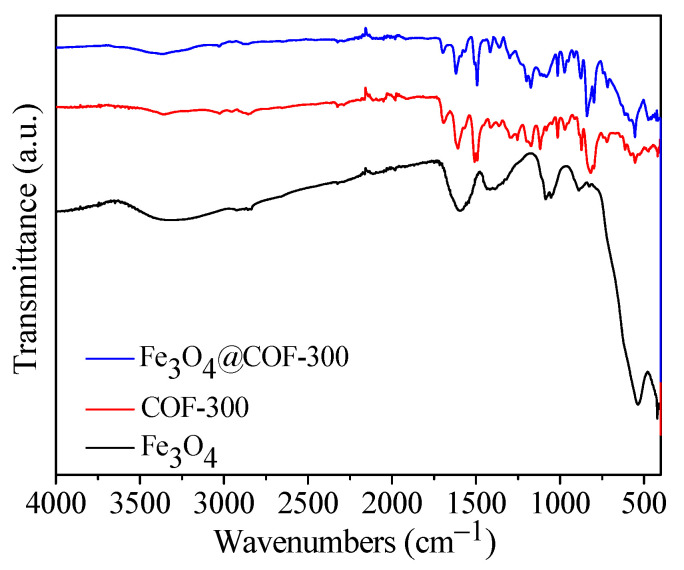
Infrared spectra of Fe_3_O_4_, COF-300 and Fe_3_O_4_@COF-300.

Fourier transform infrared (FTIR) characterization of Fe_3_O_4_, COF-300, and Fe_3_O_4_@COF-300 composites is shown in Figure 5. The main absorption peak of Fe_3_O_4_ is located at 554 cm^−1^; those of COF-300 are at 1697, 1619, 1493, and 1173 cm^−1^; and those of Fe_3_O_4_@COF-300 are at 1697, 1619, 1493, 1173, 1088, 839, and 554 cm^−1^. The absorption peak at 1697 cm^−1^ is caused by the stretching vibration of the C=O group, and that at 1619 cm^−1^ is caused by the tensile vibration of the imine C=N formed in COF-300 and Fe_3_O_4_@COF-300. The absorption peak at 1493 cm^−1^ is caused by the stretching vibration of the aromatic ring, which is consistent with the conclusion that the molecular structure of COF-300 contains an aromatic ring [23]. The new material Fe_3_O_4_@COF-300 is also absorbed at 1493 cm^−1^, indicating that COF-300 does not destroy the aromatic structure of COF-300 after recombining with Fe_3_O_4_. This finding is consistent with the XPS characterization results. The absorption peaks at 1173 and 1088 cm^−1^ correspond to the C–C telescopic vibration and Si–O–C stretching, respectively, and that at 839 cm^−1^ is an aromatic ring extended by tetraphenylmethane [16,21]. Fe_3_O_4_@COF-300 exhibits a significant absorption peak at 554 cm^−1^, which is mainly caused by the telescopic vibration of the Fe–O bond [24].

### 2.2. Adsorption Experiment

#### 2.2.1. Effect of pH on Removal Efficiency

**Figure 6:**
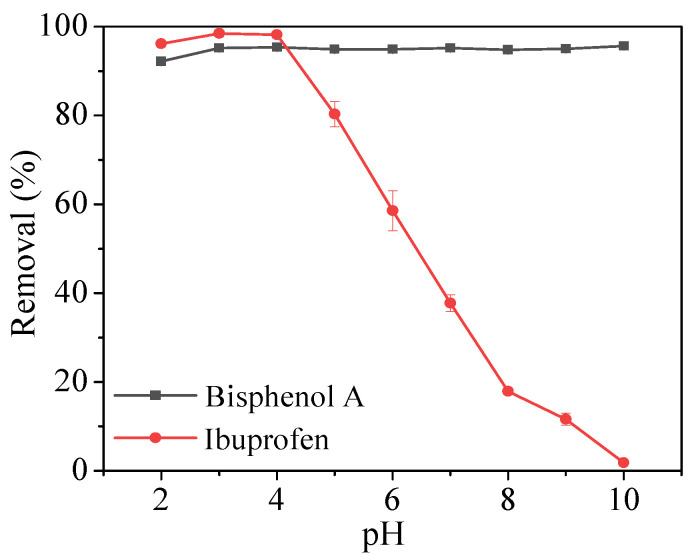
Effect of pH on bisphenol A and ibuprofen removal.

The pH of the solution was adjusted between 2 and 10 to investigate the effect of pH on the adsorption properties of bisphenol A and ibuprofen by Fe_3_O_4_@COF-300. As shown in Figure 6, the removal efficiency of bisphenol A in the pH range of 2–10 is basically unchanged and can reach more than 92.18%. For ibuprofen, when the pH was 2–4, Fe_3_O_4_@COF-300 showed good adsorption performance (≥96.12%) with a maximum removal efficiency of 98.52%. However, with the increase in pH, the removal efficiency of ibuprofen decreased continuously until it was close to zero. The reason may be that when the solution pH was higher than the acidity coefficient (p*K*_a_ = 4.91) of ibuprofen, both ibuprofen and Fe_3_O_4_@COF-300 were negatively charged (Figure S1). At such pH conditions, electrostatic repulsion occurred between the ibuprofen and the adsorption material, and the phenomenon increased with the increase in pH, thus resulting in a decrease in ibuprofen adsorption. When the solution pH was lower than the p*K*_a_ of ibuprofen, the surface of Fe_3_O_4_@COF-300 remained negatively charged (Figure S1), whereas ibuprofen was a charge-neutral molecule in water. However, the dominant dispersive interactions explained the satisfactory adsorption capacity [25]. Therefore, the adsorption of bisphenol A by Fe_3_O_4_@COF-300 was largely unaffected by pH, but the suitable pH range for ibuprofen was between 2 and 4.

#### 2.2.2. Adsorption Kinetics

**Figure 7:**
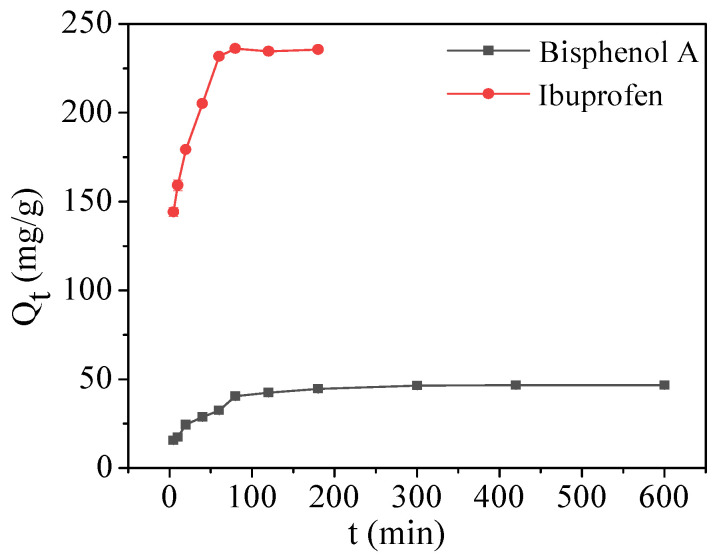
Adsorption kinetics of bisphenol A and ibuprofen by Fe_3_O_4_@COF-300.

The initial concentrations of bisphenol A and ibuprofen were set to 10 and 50 mg/L to study the adsorption kinetics. Adsorption was conducted at a certain time (5, 10, 20, 40, 60, 120, 180, 240, 300, 480, and 600 min) to determine the contaminant concentration. As shown in Figure 7, the adsorption capacity of bisphenol A and ibuprofen by Fe_3_O_4_@COF-300 increased rapidly within 60 min, reaching 69.4% and 98.2% of the equilibrium adsorption capacity, respectively. After a slow ascension, bisphenol A reached equilibrium at 120 min and ibuprofen reached equilibrium at 80 min.

The adsorption kinetic processes of bisphenol A and ibuprofen were investigated using a pseudo-second-order kinetic model (supplied in the supporting information). As shown in Figure S2 and Table S1, the adsorption processes of bisphenol A and ibuprofen were consistent with the pseudo-second-order kinetic, and there was a good linear correlation (*R*^2^ > 0.999).

#### 2.2.3. Adsorption Isotherms

**Figure 8:**
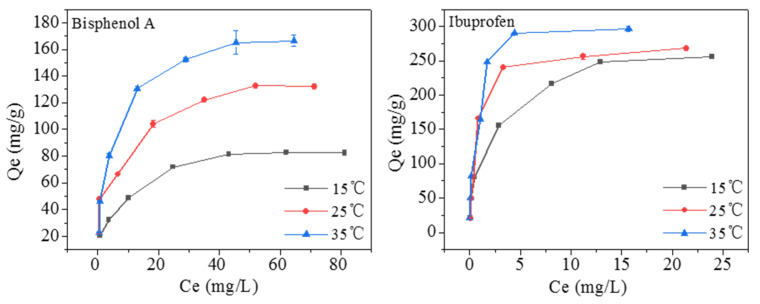
Adsorption isotherms of bisphenol A and ibuprofen.

The adsorption isotherms were studied at 15, 25, and 35 °C with the initial concentrations of bisphenol A and ibuprofen at 5–100 and 5–75 mg/L, respectively (Figure 8). The adsorption capacity of Fe_3_O_4_@COF-300 rapidly increases with the raising of the concentrations of bisphenol A and ibuprofen. At 15 °C, the adsorption capacity increases from 20.82 to 80.74 mg∙g^−1^ as the concentration of bisphenol A increases from 5 to 100 mg∙L^−1^, and it increases from 20.83 to 255.58 mg∙g^−1^ with the concentration of ibuprofen increased from 5 to 75 mg∙L^−1^. This may be due to the fact that high concentration gradients can accelerate the diffusion of pollutants into the adsorbent [26]. Meanwhile, the adsorption process is favored by high temperatures. When the concentration of bisphenol A is 40 mg∙L^−1^, the adsorption capacity of Fe_3_O_4_@COF-300 is 130.72 mg∙g^−1^ at 35 °C, and it is 104.04 and 71.87 mg∙g^−1^ at 25 and 15 °C, respectively. For ibuprofen (50 mg∙L^−1^), the adsorption capacity is 248.40 mg∙g^−1^ at 35 °C, and 240.20 and 216.26 mg∙g^−1^ at 25 and 15 °C, respectively. This result indicates that adsorption is an endothermic process.

The adsorption of bisphenol A and ibuprofen by Fe_3_O_4_@COF-300 was further analyzed using Langmuir and Freundlich models (supplied in the supporting information). Table S2 shows that for the adsorption of bisphenol A and ibuprofen, the linear correlation coefficients (*R*^2^) of the Langmuir model are 0.9937–0.9986 and 0.9963–0.9991, respectively, and those of the Freundlich model are 0.8860–0.9738 and 0.8360–0.9646, respectively. Therefore, the adsorption is consistent with the Langmuir model. Meanwhile, the maximum adsorption of bisphenol A and ibuprofen by Fe_3_O_4_@COF-300 is 173.31 and 303.03 mg·g^–1^, respectively, which is basically higher than the adsorption of other materials in the literature (Table 1). This finding shows that Fe_3_O_4_@COF-300 is of great application potential in environmental pollution control and water purification.

The thermodynamic parameters (supplied in the supporting information) of the adsorption’s progress are shown in Table 2. The adsorption capacity and Gibbs free energy change (Δ*G^θ^*) both decrease with the increase in temperature, indicating that the adsorption is an endothermic process. This result is consistent with that of the adsorption isotherm, and the positive values of enthalpy change (Δ*H^θ^*) and entropy change (Δ*S^θ^*) further confirm this conclusion. Finally, the negative value of Δ*G^θ^* indicates that the adsorption of bisphenol A and ibuprofen by Fe_3_O_4_@COF-300 is a spontaneous process.

#### 2.2.4. Effect of Ionic Strength

**Figure 9:**
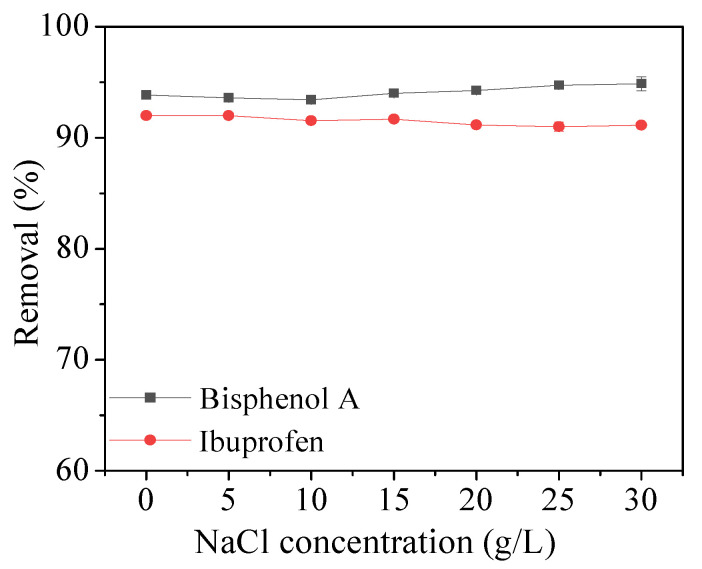
Effect of ionic strength on bisphenol A and ibuprofen removal.

The effect of ionic strength on the removal efficiency of bisphenol A and ibuprofen was analyzed by adding NaCl to control the salt concentration from 0% to 35% (*w*/*v*). Figure 9 shows that the removal efficiency of bisphenol A and ibuprofen kept stable with the increase in ionic strength. Therefore, the adsorption capacity of Fe_3_O_4_@COF-300 is unaffected by the ionic strength.

### 2.3. Selective Adsorption

**Figure 10:**
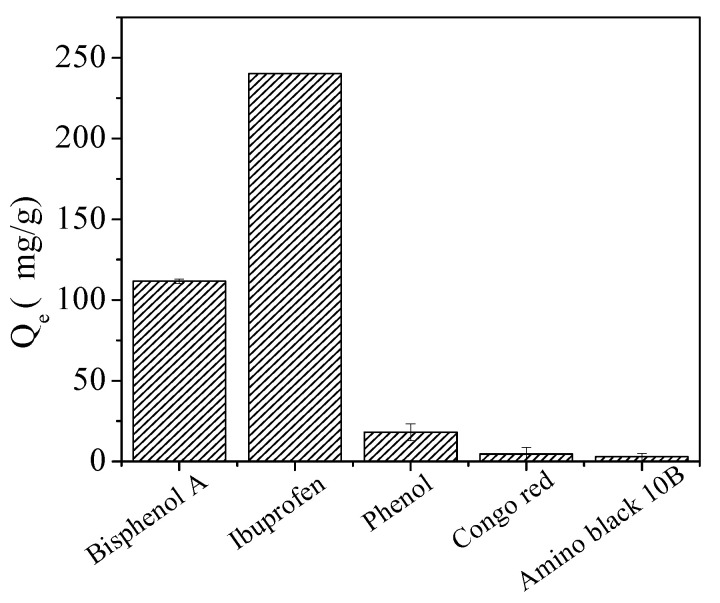
Selective adsorption capacities of Fe_3_O_4_@COF-300.

The selective adsorption properties of Fe_3_O_4_@COF-300 for bisphenol A and ibuprofen were investigated by using other coexisting pollutants commonly found in water bodies, such as phenol, Congo red, and amino black 10B. The concentrations of the coexisting pollutants were 50 mg·L^−1^, and pH was adjusted to 4.0 with HCl (0.1 mol·L^−1^). As shown in Figure 10, the adsorption of bisphenol A and ibuprofen can reach 116.68 and 240.19 mg·g^−1^, respectively, which is higher than that of phenol (18.12 mg·g^−1^), Congo red (4.59 mg·g^−1^), and amino black 10B (2.89 mg·g^−1^).

Further calculations indicate that the selectivity coefficients (supplied in the supporting information) of bisphenol A, ibuprofen, phenol, Congo red, and amino black 10B are 2.26, 93.14, 0.11, 0.01, and 0.03, respectively. These results demonstrate that Fe_3_O_4_@COF-300 exhibits high adsorption selectivity for bisphenol A and ibuprofen.

### 2.4. Environmental Implications

The adsorption behavior of Fe_3_O_4_@COF-300 on bisphenol A and ibuprofen in actual water was investigated using lake and tap water samples. The concentrations of bisphenol A and ibuprofen added to the actual water were 5–100 and 5–75 mg·L^−1^, respectively (Figure S3 and Table S3). Similar to that in pure water, the adsorption of bisphenol A and ibuprofen still conformed to the Langmuir model in lake and tap water samples. The maximum adsorption capacity of bisphenol A in lake water and tap water slightly increased by 7.47 (5.76%) and 3.46 mg·g^–1^ (2.67%), and that of ibuprofen decreased by 5.71 (2.13%) and 2.61 mg·g^–1^ (0.97%), respectively, which was less variable than that in pure water. This finding implies that Fe_3_O_4_@COF-300 has certain anti-interference characteristics and can be applied in the removal of bisphenol A and ibuprofen in actual water bodies.

### 2.5. Regeneration Property of Fe_3_O_4_@COF-300

The regeneration property of an adsorbent is a key factor in its practical application. Hence, cyclic experiments were carried out to investigate the regeneration property of Fe_3_O_4_@COF-300. Here, bisphenol A (10 mg·L^−1^) and ibuprofen (50 mg·L^−1^) were adsorbed by Fe_3_O_4_@COF-300 (200 mg·L^−1^), then the used Fe_3_O_4_@COF-300 was collected and washed 3 times with a mixture of ethanol and water (7:3, *v*/*v*), and continuously used for the removal. As shown in Figure S4, the removal efficiency of bisphenol A and ibuprofen decreased slightly with the increase in cycles. The removal efficiency of bisphenol A was 96.16% after the first cycle, and slightly decreased to 94.46%, 93.19%, and 93.02%, respectively, after the second, third, and fourth cycles. The removal efficiency could still be maintained at 92.15% after the fifth cycle. For ibuprofen, the removal efficiency was 93.37%, 92.18%, 91.24%, 89.07%, and 89.29% for the 5 cycles, respectively. Therefore, Fe_3_O_4_@COF-300 has satisfactory stability and regeneration property.

### 2.6. Adsorption Mechanism

The adsorption mechanisms of COFs for organic pollutants mainly include electrostatic and non-electrostatic interactions. Therefore, the adsorption mechanism of Fe_3_O_4_@COF-300 for bisphenol A and ibuprofen is summarized as follows.

When the solution pH is lower than the p*K*_a_ of ibuprofen, ibuprofen exist under their neutral form and Fe_3_O_4_@COF-300 is negatively charged (Figure S1). Hence ibuprofen can be adsorbed by Fe_3_O_4_@COF-300 through dispersive interactions (non-electrostatic interactions). However, when the solution pH is higher than the p*K*_a_ of ibuprofen, both ibuprofen and Fe_3_O_4_@COF-300 are negatively charged (Figure S1), and electrostatic repulsion (electrostatic interactions) occurs between them [25].

Bisphenol A is minimally affected by the solution pH, so the main adsorption mechanism is non-electrostatic. (1) As indicated by the FTIR results, Fe_3_O_4_@COF-300 has an aromatic structure, while bisphenol A also contains aromatic structures as well as abundant π electrons. Due to the π-π bond interactions between aromatic compounds, bisphenol A can undergo π-π interactions with the rich conjugated π domains in Fe_3_O_4_@COF-300 [37]. (2) Both Fe_3_O_4_@COF-300 and bisphenol A are hydrophobic, hence, hydrophobic interactions may also occur between them [38]. (3) Fe_3_O_4_@COF-300 composites are rich in N-containing functional groups (C=N−C) that may form hydrogen bonds with hydroxyl groups in bisphenol A, thus increasing the intermolecular forces and facilitating the adsorption [39].

## 3. Materials and Methods

### 3.1. Chemicals and Reagents

Bisphenol A, ibuprofen, phenol, Congo red, and amino black 10B were purchased from J&K Scientific Co., Ltd., Beijing, China. Tetrakis (4-aminophenyl) methane was purchased from Maclean Biochemical Technology Co. Ltd., China. All other reagents were of reagent grade and obtained from Bodi Chemical Industry Co., Ltd., Chongqing, China.

### 3.2. Preparation of Fe_3_O_4_@COF-300

Crushed FeCl_3_·6H_2_O (1.39 g) was dissolved in 75 mL of ethylene glycol. Crushed sodium acetate solids (3.56 g) were then added to the solution, sealed with fresh-keeping film, and continuously blended with a magnetic stirrer at room temperature for 1 h to form a uniform mixed solution. The mixed solution was then sonicated for 15 min to dissolve all the solids, transferred to a Teflon-lined stainless autoclave and heated at 200 °C for 8 h. The product was washed with ethanol 3 times after cooling down and maintained at 60 °C for 6 h. The final Fe_3_O_4_ product was obtained.

In order to prevent the reaction between Fe_3_O_4_ and acetic acid, as well as to prevent the oxidation and aggregation of Fe_3_O_4_, Fe_3_O_4_ was first compounded with SiO_2_ (Fe_3_O_4_@SiO_2_). The prepared Fe_3_O_4_ (100 mg) dispersed in ethanol (150 mL), ammonia (3 mL) and deionized water (47 mL) was added sequentially to the suspension, and the mixture (ethanol:deionized water:ammonia, 75.0%:23.5%:1.5%, *v*:*v*:*v*) was sonicated for 40 min. Ethyl orthosilicate (0.6 mL) was then added at room temperature, stirring was continued for 8 h. Finally, the product was collected by magnet, washed several times with ethanol and dried under vacuum at 60 °C.

Fe_3_O_4_@SiO_2_ (0.1 g), 1,4-phthalaldehyde (0.25 g), tetrakis (4-aminophenyl) methane (0.4 g), 1,4-dioxane (25 mL) and acetic acid solution (4 mL, 3 mol/L) were mixed. After sonication, the above mixture was transferred to a Teflon-lined autoclave and then heated at 120 °C for 72 h. Finally, the composite was washed alternately with 1,4-dioxane and tetrahydrofuran 3 times, and Fe_3_O_4_@COF-300 was obtained after drying at 60 °C.

### 3.3. Adsorption Experiment

Batch adsorption experiments were performed in a glass reactor with a volume of 25 mL. The volume of the reaction solution was 15 mL with a certain amount of bisphenol A and ibuprofen and 0.2 g·L^−1^ of Fe_3_O_4_@COF-300. The pH was adjusted with HCl and NaOH (0.1 mol/L). The mixture was placed in a water bath shaker at 25 °C for 12 h to completely balance the reaction. The reaction solution was placed on a strong magnet for 5 min after adsorption, and 1 mL of the supernatant was then extracted for contaminant concentration analysis. All the experiments were conducted twice to ensure the reproducibility and reliability of the results.

### 3.4. Analysis Methods

SEM images were acquired using a SUPPA 55 scanning electron microscope (Zeiss, Germany). XRD patterns were conducted with a D/max-r8 diffractometer (Rigaku, Akishima, Japan). XPS were obtained by an ESCALAB Xi+ system (Thermo Fisher Scientific, Waltham, MA, USA). FTIR spectra were recorded by Mangna–550 (Thermo Nicolet Corporation, Edina, MN, USA). TGA curves were obtained using an SDT Q600 thermogravimeter at 0–800 °C (TA Instruments, New Castle, DE, USA). Zeta potential analysis was measured by a potential analyzer (Malvern Zetasizer Nano ZS90, Marvin, UK). Bisphenol A, ibuprofen, and phenol concentrations were determined by an UltiMate3000 high-performance liquid chromatograph (HPLC; Thermo Fisher Scientific, Waltham, MA, USA). Congo red and amino black 10B concentrations were determined by a T6 UV-vis spectrophotometer (Beijing Purkinje, Beijing, China).

### 3.5. Preparation of Simulated Water Samples

A certain amount of bisphenol A and ibuprofen was added to the actual water sample to prepare the simulated water sample and explore the adsorption performance of Fe_3_O_4_@COF-300. All actual water samples were obtained from Yantai in Shandong Province. Tap water samples were taken from a laboratory of the Chemistry Building at Ludong University (Yantai, China). Lake water samples were acquired from the campus of Ludong University (Yantai, China). All samples were randomly collected and filtered to remove suspended particles and then used immediately within 24 h.

## 4. Conclusions

In this study, magnetic composites Fe_3_O_4_@COF-300 were successfully prepared and adopted for the adsorption of bisphenol A and ibuprofen in water. The composites could not only be rapidly separated from the matrix by external magnetic fields, but also showed excellent selective adsorption capacity for bisphenol A and ibuprofen. The adsorption process conformed to the pseudo-second-order kinetic and Langmuir model. Fe_3_O_4_@COF-300 presented satisfactory anti-interference characteristics, stability, and regeneration property and realized the adsorption of ibuprofen through electrostatic and non-electrostatic interactions, and bisphenol A through π–π interaction, hydrophobic interaction, and the formation of hydrogen bond. Fe_3_O_4_@COF-300 could be applied for the efficient removal of bisphenol A and ibuprofen in actual water bodies.

## Figures and Tables

**Table 1:** Adsorption capacity of bisphenol A and ibuprofen on reported sorbents.

Adsorbates	Adsorbents	Q_max_ (mg·g^–1^)	References
Bisphenol A	nZVI–chitosan	65.16	[27]
	modified carbon nanotubes	69.93	[28]
	β-Cyclodextrin-modified graphene oxide membranes	25.50	[29]
	modified montmorillonite (BS–Mt)	80.77	[30]
	β-Cyclodextrin/ZrO_2_	174.90	[31]
Ibuprofen	ZIF–8 derived porous carbon	320.00	[32]
	NiFe_2_O_4_/AC	261.35	[33]
	MAP	269.61	[34]
	chitosan modified rubber	70.00	[35]
	cysteine-modified silane-coated magnetic nanomaterial	138.10	[36]

**Table 2:** Thermodynamic parameters of bisphenol A and ibuprofen.

Pollutant	*C*_0_ (mg/L)	*C_e_* (mg/L)			Δ*S^θ^* (J/mol/K)	Δ*H^θ^* (kJ/mol)	Δ*G^θ^* (kJ/mol)		
		288 K	298 K	308 K			288 K	298 K	308 K
Bisphenol A	100	81.65	71.86	64.99	120.62	34.69	−0.07	−1.27	−2.48
Ibuprofen	75	23.90	21.39	15.68	92.07	20.96	−24.43	−25.35	−26.28

## Data Availability

All data generated or analyzed during this study are included in this published article.

## Supplementary Materials

The following supporting information can be downloaded at: https://www.mdpi.com/article/10.3390/molecules28135214/s1, Figure S1: Zeta potential analysis of Fe_3_O_4_@COF-300; Figure S2: Adsorption kinetics of bisphenol A and ibuprofen by Fe_3_O_4_@COF-300; Figure S3: Adsorption isotherms of bisphenol A and ibuprofen in actual water samples; Figure S4: Regeneration property of Fe_3_O_4_@COF-300; Table S1: Pseudo-second-order reaction kinetics fitting of bisphenol A and ibuprofen removal with Fe_3_O_4_@ COF-300; Table S2: Parameters of Langmuir and Freundlich models for bisphenol A and ibuprofen with Fe_3_O_4_@COF-300; Table S3: Langmuir and Freundlich isotherms parameters of bisphenol A and ibuprofen in actual water samples.

## Sample Availability

Samples of all compounds are available from the authors.
