# Supplementary material for: Adsorption Performance of Magnetic Covalent Organic Framework Composites for Bisphenol A and Ibuprofen
Source: Molecules. 2023 Jul 5;28(13):5214. doi: 10.3390/molecules28135214 (PMC10343673; doi:10.3390/molecules28135214)
Supplement: Supplementary file 1 [file molecules-28-05214-s001.zip › molecules-2483721-supplementary.pdf]

Supplementary Material for

Adsorption performance of magnetic covalent organic framework composites for bisphenol A and ibuprofen

Adsorption kinetics

The pseudo second-order kinetic model was applied to describe the adsorption kinetic and its linear model is expressed as follows:

$$\frac{t}{Q_t} = \frac{1}{KQ_e^2} + \frac{1}{Q_e}t \quad (1)$$

Where K (g/mg/min) is the rate constant of adsorption, Q_t (mg/g) is the adsorption amount of dye at any time, and Q_e (mg/g) is the equilibrium adsorption capacity. The initial adsorption rate h (mg/g/min) can be defined as follows:

$$h = KQ_e^2 (t \rightarrow 0) \quad (2)$$

Adsorption isotherms

Adsorption data of bisphenol A and ibuprofen by Fe_3O_4@COF-300 were analyzed with the Langmuir and Freundlich isotherm models as follows:

$$\frac{C_e}{Q_e} = \frac{1}{\theta b} + \frac{C_e}{\theta} \quad (3)$$

$$ln Q_e = ln K_F + \frac{1}{n} ln C_e \quad (4)$$

Where Q_e ($mg \cdot g^{-1}$) and C_e ($mg \cdot L^{-1}$) are the equilibrium adsorption capacity and the equilibrium concentration, respectively; θ ($mg \cdot g^{-1}$) is the maximum adsorption capacity; b ($L \cdot mg^{-1}$) is the equilibrium adsorption constant; and K_F ($mL^{1/n} \mu g^{1-1/n}$) and

n are Freundlich constants. Values of K_F and n can be obtained from the linear quasi-cooperative graph of lnQ_e and lnC_e, respectively.

Adsorption thermodynamics

Thermodynamic parameters can be obtained from the slope of the linear plot of lnK_c versus $1/T$ as follows:

$$K_c = \frac{Q_e}{C_e} \tag{5}$$

$$ln\,K_c = \frac{\Delta S^\theta}{R} — \frac{\Delta H^\theta}{RT} \tag{6}$$

$$\Delta G^\theta = \Delta H^\theta — T\Delta S^\theta \tag{7}$$

where Q_e (mg·g^{-1}) and C_e (mg·L^{-1}) are the equilibrium adsorption capacity and the equilibrium concentration, respectively; K_c and T are the distribution coefficient and solution temperature, respectively; and R is the gas constant (8.314 J·mol^{-1}·K^{-1}). ΔG^θ (KJ·mol^{-1}), ΔH^θ (KJ·mol^{-1}), and ΔS^θ (J·mol^{-1}·K^{-1}) are the respective changes in Gibbs free energy, enthalpy, and entropy under standard states.

The value of ΔH^θ and ΔS^θ can be obtained from the slope of the linear plot of lnK_c versus $1/T$. ΔG^θ can be obtained using Eq. (7).

Selectivity coefficient

The selection coefficient is used to express the selective adsorption of pollutants by the adsorbents.

Selection coefficient = (mass fraction of the target pollutant adsorbed by adsorbent/ mass fraction of other pollutants adsorbed by adsorbent)/(mass fraction of the target

pollutant remaining in the solution /mass fraction of other pollutants remaining in the solution).

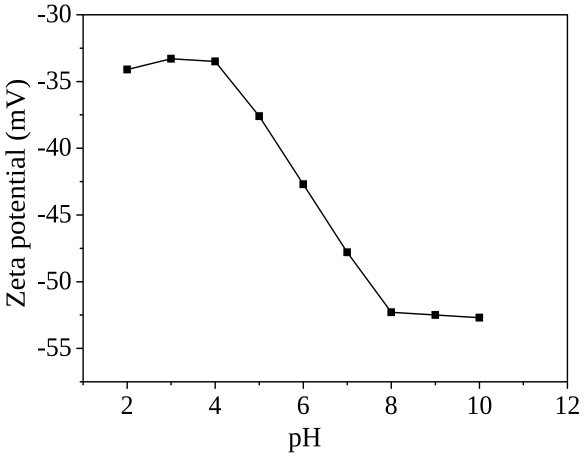

Figure S1. Zeta potential of Fe_3O_4@COF-300.

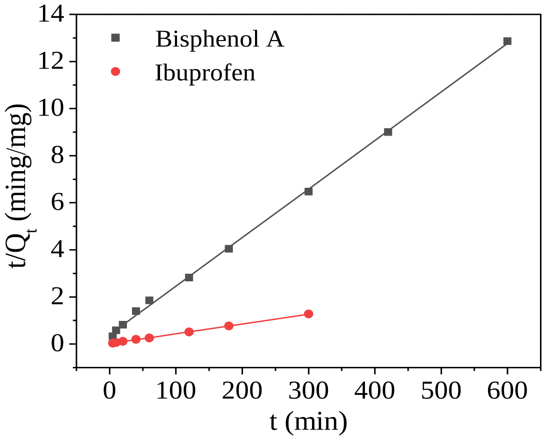

Figure S2. Adsorption kinetics of bisphenol A and ibuprofen by Fe_3O_4@COF-300.

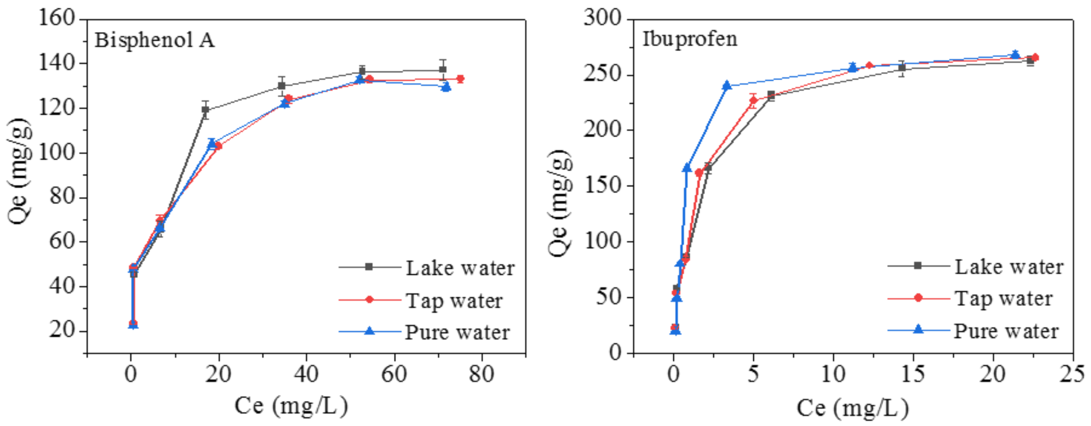

Figure S3. Adsorption isotherms of bisphenol A and ibuprofen in actual water samples.

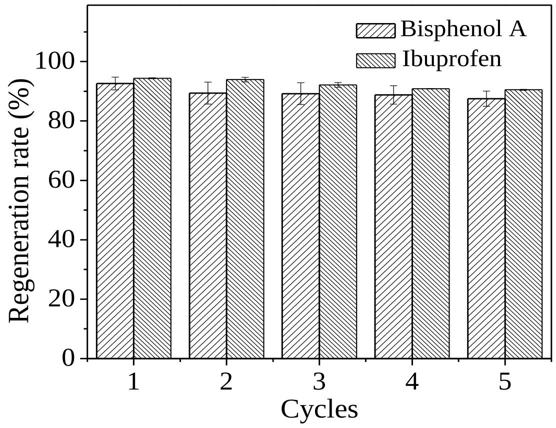

Figure S4. Regeneration property of Fe_3O_4@COF-300.

Table S1. Pseudo-second-order reaction kinetics fitting of bisphenol A and ibuprofen removal with Fe_3O_4@ COF-300.

Pollutant	K (g/mg/min)	h (mg/g/min)	R^2
Bisphenol A	1.07×10^{-3}	2.513	0.9991
Ibuprofen	9.24×10^{-4}	53.135	0.9997

Table S2. Parameters of Langmuir and Freundlich models for bisphenol A and ibuprofen with Fe_3O_4@COF-300.

Pollutant	Temperature (°C)	Langmuir equation			Freundlich equation		
		θ (mg·g^{-1})	b (g·mL^{-1})	R^2	K_F (m L$^{1/n}$·μg$^{1-1/n}$)	n	R^2
Bisphenol A	15	87.64	0.194	0.9965	22.479	3.105	0.9738
	25	137.36	0.258	0.9937	39.865	3.338	0.8860
	35	173.31	0.301	0.9986	42.423	2.707	0.9367
Ibuprofen	15	266.67	0.813	0.9963	87.985	2.465	0.9646
	25	278.55	1.189	0.9991	102.382	2.300	0.8360
	35	303.03	2.920	0.9978	159.913	3.272	0.9596

Table S3. Langmuir and Freundlich isotherms parameters of bisphenol A and ibuprofen in actual water samples.

Pollutant	Samples	Langmuir equation			Freundlich equation		
		θ (mg·g^{-1})	b (g·mL^{-1})	R^2	K_F (mL$^{1/n}$·μg$^{1-1/n}$)	n	R^2
Bisphenol A	Lake water	144.30	0.269	0.9943	39.519	3.118	0.9244
	Tap water	139.86	0.240	0.9944	39.199	3.267	0.9206
Ibuprofen	Lake water	277.78	0.759	0.9988	88.716	2.310	0.9209
	Tap water	279.33	0.867	0.9988	93.660	2.332	0.9158
